# AI-based shade matching versus visual assessment: Accuracy in dental aesthetics

**DOI:** 10.6026/973206300221306

**Published:** 2026-03-31

**Authors:** Vijay Shekhar, Gurmehak Kaur Sandhu, Jaskiran Kaur Nain, Vrushali Vasant Bhoir, Sneha Das, Yihan Fu, Heena Dixit Tiwari

**Affiliations:** 1 Department of Dentistry, Nalanda Medical College Hospital, Agamkuan, Patna, Bihar, India; 2 Luxmi Bai Institute of Dental Sciences and Hospital, Sirhind Road, Patiala, Punjab, India; 3 Department of Periodontology, Dr. D. Y. Patil Dental College and Hospital, Pimpri, Pune, Maharashtra, India; 4 Department of Public Health Dentistry, Late Shri Yashwantrao Chavan Memorial Medical & Rural Development Foundation's Dental College & Hospital, Ahmednagar, Maharashtra, India; 5 General Dentist, Smilebuilderz, Lancaster, Pennsylvania, United States; 6 Programme Officer, Blood Cell, Commissionerate of Health and Family Welfare, Government of Telangana, Hyderabad, India

**Keywords:** Artificial intelligence, color perception, dental esthetics, tooth color, colorimetry

## Abstract

Visual shade selection in dentistry is inherently subjective and influenced by lighting conditions and examiner variability. Artificial intelligence (AI)-based shade matching systems provide objective, data-driven color analysis to improve consistency in aesthetic dentistry. This study compared AI-based shade matching with conventional visual assessment using a spectrophotometer as the reference standard. Sixty patients were evaluated using VITA (Vereinigte Internationale für Technologie in der Zahnmedizin) shade guides, AI software, and spectrophotometric measurements. AI achieved higher accuracy (86.7%) than visual assessment (58.3%) with statistical significance (p=0.031). Reproducibility was greater with AI (κ=0.78) compared to visual methods (κ=0.42). AI-based shade matching demonstrated superior accuracy and consistency, reducing examiner bias. Integration of AI in clinical practice may improve restorative outcomes, laboratory communication, and patient satisfaction

## Background:

Dental aesthetics strongly influence patient satisfaction and clinical success, with tooth color as a key determinant. Accurate shade matching is essential for restorative and prosthetic treatments, including veneers, crowns and composite restorations [1]. Visual shade selection using commercial shade guides is common in practice but remains prone to operator subjectivity, lighting variability, fatigue and inter-observer differences [2]. Even experienced clinicians frequently disagree in shade selection, resulting in wide variability in reproducibility rates [3]. Standardized visual systems, such as VITA Classical and VITA 3D-Master shade guides, improve consistency but remain inherently subjective [4].

The demand for precise and reproducible shade selection has accelerated the adoption of digital and AI-driven technologies in dentistry [5]. AI-based shade matching integrates advanced imaging and machine learning to provide objective tooth color analysis [6]. These systems use spectral reflectance and digital calibration to minimize human and environmental bias [16]. With growing emphasis on minimally invasive aesthetic dentistry, accurate shade determination directly influences clinical outcomes, laboratory communication, and patient confidence [7]. Therefore, it is of interest to evaluate AI-based shade matching against conventional visual assessment is clinically relevant for assessing accuracy and reproducibility in dental practice.

## Materials and Methods:

## Study design:

A comparative *in vitro* and *in vivo* observational study was conducted to assess shade-matching accuracy.

## Sample:

[1] Participants: 60 patients requiring anterior restorations.

[2] Teeth assessed: Maxillary central incisors.

[3] Examiners: 10 dental practitioners with ≥3 year's clinical experience.

## Methods:

## Visual assessment:

[1] Performed using VITA Classical and VITA 3D-Master shade guides.

[2] Each examiner independently selected shades under standardized lighting (5500K).

[3] Final visual shade determined by consensus.

## AI-based assessment:

[1] Digital images captured using intraoral camera linked to AI software.

[2] AI algorithm analyzed hue, chroma and value, generating shade recommendation.

## Reference standard:

Spectrophotometer readings considered gold standard.

## Evaluation criteria:

[1] Accuracy: Match with spectrophotometer reading.

[2] Reproducibility: Agreement among examiners.

[3] Statistical analysis: Chi-square test for categorical data, kappa coefficient for inter-rater reliability, significance set at p< 0.05.

## Results and Discussion:

Table 1 (see PDF) shows the comparative accuracy of AI-based shade matching and visual assessment against the spectrophotometer reference. AI-based systems achieved 86.7% accurate matches, significantly higher than the 58.3% obtained with visual methods (p=0.031). Visual ssessment demonstrated operator-dependent bias and inconsistent interpretation, resulting in greater variability in shade selection. In contrast, AI provided consistent and reproducible color evaluation, reducing errors in aesthetic restorative procedures. Table 2 (see PDF) presents inter-examiner reproducibility using kappa statistics. Visual assessment showed moderate agreement (κ=0.42), indicating variability among clinicians despite standardized lighting conditions. AI-based systems exhibited substantial agreement (κ=0.78), reflecting superior objectivity and consistency across users. These findings confirm that AI reduces examiner-dependent discrepancies and improves predictability in clinician-laboratory communication. Shade selection remains a critical challenge in aesthetic dentistry, as inaccuracies contribute to patient dissatisfaction and prosthetic remakes [8]. The present study demonstrated significantly higher accuracy and reproducibility of AI-based systems compared with conventional visual methods. Visual shade selection is influenced by examiner color perception, age, fatigue, and ambient lighting, limiting reproducibility even under controlled conditions [9]. AI-based tools overcome these limitations by objective digital color analysis aligned with spectrophotometric standards [1]. In a prospective comparative clinical study, AI-based digital smile design demonstrated significantly higher shade-match accuracy than conventional visual methods; achieving 85% clinically acceptable matches (ΔE00 ≤1.8) compared with 70% using traditional shade selection, along with reduced chairside time and improved reproducibility [11]. AI-based shade analysis has been reported to exceed 80% accuracy, corroborating recent trials where machine learning algorithms outperformed human examiners [12]. Clinical adoption of AI improves dentist-laboratory communication and minimizes subjective discrepancies in shade transfer. AI-based documentation also enables tracking and reproducible outcomes in long-term restorative care [1]. The study has certain limitations. The modest sample size (n=60) restricts generalization of findings. Use of a single AI platform limits cross-platform comparability. *In vivo* conditions such as saliva and surface reflections may influence optical readings. Future studies involving larger populations and multiple AI systems are recommended to validate clinical applicability [14, 15].

## Conclusion:

AI-based shade matching show significantly higher accuracy and reproducibility compared to conventional visual assessment in dental aesthetics. While visual methods remain widespread due to low cost and accessibility, AI systems offer objective, standardized and reliable outcomes. Incorporating AI into routine dental practice may reduce errors, improve laboratory communication and enhance patient satisfaction. Despite initial investment challenges, the long-term benefits suggest AI will become integral in aesthetic restorative dentistry.

## References

[1] Kaushik K (2025). BDJ Open..

[2] Mohsin L (2025). J Pharm Bioallied Sci..

[3] El-Etreby A (2024). J Esthet Restor Dent..

[4] Yeslam HE (2024). Peer J..

[5] Zhu J (2024). BMC Oral Health..

[6] Manauta J (2024). J Esthet Restor Dent..

[7] Shetty S (2024). J Prosthodont..

[8] Kose C (2023). J Esthet Restor Dent..

[9] Liu CMJr (2024). Dent Mater..

[10] Tabatabaian F (2021). J Esthet Restor Dent..

[11] Shetty R (2025). Bull Stomatol Maxillofac Surg..

[12] Soares PM (2023). J Esthet Restor Dent..

[13] Segundo ARTC (2023). J Esthet Restor Dent..

[14] Sen N, Sermet B (2024). Int J Prosthodont..

[15] Fouda AM (2023). Clin Oral Investig..

